# A Canadian, retrospective, multicenter experience with selexipag for a heterogeneous group of pediatric pulmonary hypertension patients

**DOI:** 10.3389/fped.2023.1055158

**Published:** 2023-02-28

**Authors:** David Youssef, Susan Richards, Sabine Lague, Catherine Sheppard, Jenna Smith, Erika Vorhies, Martin Hosking, Matthew Pietrosanu, Angela Bates

**Affiliations:** ^1^ Department of Pediatric Pulmonary Hypertension, Stollery Children’s Hospital, Edmonton, AB, Canada; ^2^ Department of Pediatrics, BC Children’s Hospital, Vancouver, BC, Canada; ^3^ Department of Pharmacy, Stollery Children’s Hospital, Edmonton, AB, Canada; ^4^ Department of Pediatric Cardiology, Alberta Children’s Hospital, Calgary, AB, Canada; ^5^ Department of Pediatric Cardiology, BC Children’s Hospital, Vancouver, BC, Canada; ^6^ Department of Mathematical and Statistical Sciences, University of Alberta, Edmonton, AB, Canada; ^7^ Division of Pediatric Critical Care, Department of Pediatrics, University of Alberta, Edmonton, AB, Canada

**Keywords:** adverse effect, congenital heart disease, echocardiography, infant, nonprostanoid, prostaglandin receptor, side effect

## Abstract

**Introduction:**

Selexipag, an oral nonprostanoid prostaglandin receptor agonist, has led to reduced morbidity and mortality in adults with pulmonary arterial hypertension (PAH). While the adult literature has been extrapolated to suggest selexipag as an oral treatment for severe pediatric pulmonary hypertension (PH), longitudinal, multicenter data on the benefits of selexipag in this population are lacking. The purpose of this study is to present a longitudinal, multicentre experience with selexipag in a relatively large cohort of pediatric PH patients and add to the existing selexipag literature.

**Materials and methods:**

We performed a retrospective, multicenter review describing the clinical outcomes of pediatric PH patients receiving selexipag in addition to standard oral pulmonary vasodilator therapy across three Canadian centers between January 2005 and June 2021.

**Results:**

Twenty-four pediatric patients (fifteen female) with a mean age of 9.7 (range 2.0–15.5) years were included. Of this cohort, eighteen (75.0%) were in group 1, one (4.2%) was in group 2, four (16.7%) were in group 3, and one (4.2%) was in group 4. Twenty-two (91.7%) patients were on dual PH therapy after six months. Dosing was targeted to achieve 20–30 mcg/kg/dose orally every twelve hours. Median dose after twelve months was 30 mcg/kg/dose. Twelve months following selexipag initiation, median decreases of 0.2 cm in tricuspid annular plane systolic excursion, 3.5 mmHg in right-ventricular systolic pressure, and 6.1 mmHg in mean pulmonary arterial pressure were observed; none of these changes were statistically significant. Three patients died, one clinically deteriorated and required admission to a pediatric intensive care unit, ten had gastrointestinal symptoms, and three had flushing.

**Conclusion:**

Selexipag appears to be a safe and effective adjunctive therapy for pediatric PH patients and has a tolerable adverse effect profile aside from gastrointestinal disturbances. Additional prospective studies of changes in hemodynamics and functional classification over a longer period and with a larger sample are needed. Future research should aim to identify subgroups that stand to benefit from the addition of selexipag as well as optimal timing and dosing for the pediatric population.

## 1. Introduction

Pulmonary hypertension (PH) is a rare disorder with a high burden of morbidity and mortality. Estimates of incidence and prevalence are comparable across registries around the world despite their slightly different inclusion and diagnostic criteria. Historical data from two large pediatric registries (United States and Europe) suggest a PH incidence of 4–10 cases per million children per year and a prevalence of 20–40 cases per million children among the pediatric population (1). Mortality is particularly high for patients with idiopathic or heritable pulmonary arterial hypertension (PAH). Without appropriate treatment or a lung transplant, PAH is a progressive and fatal chronic disease. As such, improving patient quality of life and decreasing the risk of mortality through pulmonary vasodilators has proven key to the chronic management of pediatric PH.

Understanding of the pathophysiology of pediatric PH has grown significantly over the past decade. Collaborative efforts, most recently in 2018 through the 6th World Symposium on Pulmonary Hypertension (WSPH), have refined classification groups using evolving knowledge of this disease in the pediatric population (2, 3). The two dominant groups of pediatric PH according to this system are group 1, which includes patients with the disease at the pulmonary arterial level, and group 3, which encompasses patients with an underlying lung pathology. Along with classification systems, knowledge of hemodynamics is evolving, most notably regarding the group 1 category of the disease (Supplementary Table S1). Pulmonary vasodilators have historically been targeted at group 1 patients. However, an evolving body of experience suggests that a subset of group 3 pediatric patients benefit from these same vasodilator therapies (2–4).

Given the heterogeneity and complex etiology of pediatric PH, there is a need to expand the existing body of literature on treatment options and outcomes with combination therapy. Although extrapolation from robust adult data has driven the use of various agents to treat pediatric PH, there are major differences between adult and pediatric PH. These include characteristic differences in etiology, presenting symptoms, and acute vasodilator response (3, 4). Children with PH not only have different pathophysiologies, but also tend to have more-aggressive PH than their adult counterparts as well as unique pharmacokinetic and developmental factors. Treatment strategies for pediatric PH must take these differences into consideration.

Current treatment options for pediatric PH, as for adult PH, aim to target one of three major pathways: the endothelin, nitric oxide, and prostacyclin pathways (5, 6). Oral ERA and PDE5 inhibitor therapies respectively target the endothelin and nitric oxide pathways. Synthetic prostacyclins, which target the prostacyclin pathway, are administered either subcutaneously, orally, by inhalation, or by central venous lines (CVLs). In particular, CVL catheter care presents significant challenges for both patients and caregivers, requires sterile procedures, and is associated with higher risks of infection and thrombosis, which further contributes to the morbidity and mortality of pediatric PH (7). The availability and use of subcutaneous treprostinil, a prostacyclin therapy that has improved mortality among pediatric patients in group 1 and has benefits similar to epoprostenol, has lowered the risks associated with CVLs, slowed disease progression, and improved clinical outcomes. However, dermatitis and site pain are very common adverse effects of subcutaneous treprostinil (8).

Sitbon et al. (9) reported that triple-upfront, PH-targeted therapy benefits adult patients with severe PH; their multicenter, double-blind, placebo-controlled phase 3 study showed that, for group 1 adults, selexipag significantly decreases complications and death related to PH. Although oral and inhaled forms of prostacyclin were available in other regions, until selexipag, an oral nonprostanoid prostaglandin receptor agonist, was approved by Health Canada in January 2016, in Canada, treatments acting on the prostacyclin pathway required continuous subcutaneous or intravenous infusion. The addition of an oral prostaglandin receptor agonist was a welcome addition in the early management of aggressive adult PH, but until recently its utility in the pediatric population was limited to regions or countries with access. Within Canada, both out of necessity and availability, the use of selexipag across pediatric PH groups is growing. This oral option offers more-aggressive therapy earlier in the course of the disease and significantly improves patient quality of life. However, given the lack of evidence in this particularly vulnerable population, clinical conditions and the course of the disease need to be followed closely.

Despite the growing collective experience with selexipag for treating pediatric PH (10–14), present literature on the clinical benefits of this drug is scarce, especially with regards to the younger population (Supplementary Table S1). The largest study of pediatric PH is a multicenter case report series from Germany that describes experiences with selexipag in patients with WHO functional classifications of III and IV. Most other data on the benefits and side effects of selexipag come from a small number of case reports. No multicenter research on selexipag outcomes in the pediatric population exists in the present literature.

The purpose of this study is to examine the clinical outcomes of pediatric patients with PH who receive selexipag therapy in addition to standard pulmonary vasodilator therapy. We anticipate that selexipag is a beneficial adjunctive therapy for pediatric PH and can help improve quality of life and clinical measures.

## 2. Materials and methods

We present a retrospective study of pediatric patients with PH who were prescribed selexipag therapy. We characterized clinical progression while on selexipag through close clinical follow-ups, biochemical markers, echocardiographic examinations, cardiac catheterization hemodynamics, the six-minute walk test, dosing patterns, and drug tolerance.

Patients between 0 and 18 years of age with a PH diagnosis who were managed at one of three participating pediatric PH centers in Western Canadian and who had been prescribed selexipag by their PH specialists were included. Data from the Stollery Children's Hospital, Alberta Children's Hospital, and BC Children's Hospital between January 1, 2005 and June 30, 2021 were used in this study.

Assessments were performed at baseline, selexipag initiation, and six and twelve months after initiation. Demographic and clinical data were collected for all patients at baseline. Anthropometric data, cardiac catheterization data, echocardiographic data, and clinical measures were collected at each time point.

This work was approved by the Health Research Ethics Board at the University of Alberta (approval number Pro00084720).

Statistical analyses were conducted using R version 3.6.3 (15). We present descriptive statistics to summarize patient demographics and clinical characteristics at each time point. Comparisons between baseline and twelve months are conducted with paired Wilcoxon signed-rank tests with a threshold significance of 0.05. Raw *p*-values (i.e., without adjustment) are presented throughout.

## 3. Results

Of the twenty-four patients included in this study, fifteen (62.5%) were female. The median age at initiation was 9.7 (range 2.0–15.5) years. Eighteen (75.0%) patients were in group 1, one (4.2%) was in group 2 (4.2%), four (16.7%) were in group 3, and one (4.2%) was in group 4. Among all patients, seventeen (70.8%) had underlying congenital heart disease (CHD) and five (20.8%) had obstructive sleep apnea. Trisomy 21 was present in four (16.7%) patients and DiGeorge syndrome in one (4.2%). Comorbidities varied among the patients: two (8.3%) were preterm infants, three (12.5%) had underlying respiratory diseases, five (20.8%) had ENT issues, two (8.3%) had musculoskeletal issues, and one (4.2%) had neurological issues. Summaries of these and other demographic and clinical characteristics are provided in Table 1; see Supplementary Table S2 for more-detailed comorbidity classifications. At the initiation of therapy, median patient weight was 28.4 (*Q*_1_ = 24.4, *Q*_3_ = 37.7) kg and median height was 136.0 (*Q*_1_ = 112.9, *Q*_3_ = 149.6) cm. Anthropometric data over the course of the study period are summarized in Supplementary Table S3.

**Table 1:** Demographic and clinical characteristics at baseline and comorbidities at selexipag initiation, presented as median (*Q*_1_, *Q*_3_) or count (%).

Variable/level	Summary
**Demographics**	
Sex: female	15 (62.5%)
Sex: male	9 (37.5%)
Age (years)	9.7 (7.5, 11.0)
**WSPH classification (code) at diagnosis**	
Idiopathic PAH (1.1)	8 (33.3%)
BMPR2 mutation (1.2.1)	1 (4.2%)
PAH associated with congenital heart disease (1.4.4)	9 (37.5%)
PH due to left heart disease (2)	1 (4.2%)
PAH due to lung disease (3)	4 (16.7%)
PAH due to chronic pulmonary artery obstruction (4)	1 (4.2%)
**Comorbidities** **^1^**	
Asthma	1 (4.2%)
Clotting disorder	2 (8.3%)
Congenital heart disease	17 (70.8%)
Obstructive sleep apnea	5 (20.8%)
Other	14 (58.3%)
None	1 (4.2%)
**Genetic syndromes**	
DiGeorge syndrome	1 (4.2%)
Noonan syndrome	0 (0.0%)
Russell–Silver syndrome	0 (0.0%)
Trisomy 21	4 (16.7%)
Other	2 (8.3%)

^1^ Refer to Supplementary Table S2 for a further breakdown.

Despite initial improvements, three patients died. One of these patients was in group 2 with a background of critical aortic stenosis and underwent balloon valvuloplasty, but showed evidence of endocardial fibroelastosis. This patient ultimately received a left-ventricular assistance device, fared well on oral selexipag, but died from complications secondary to a HeartMate III pump exchange. The second patient was suspected to have capillary hemangiomatosis, but died from an underlying immune deficiency and chronic interstitial lung disease. The third patient, who was born prematurely and had trisomy 21, had a complete atrioventricular septal defect and Eisenmenger physiology, and died from a secondary viral infection prior to prostacyclin therapy.

Supplementary Table S4 summarizes changes in the therapies received by the cohort. At twelve months, twenty-two (91.7%) patients were on dual pulmonary vasodilator therapy. At baseline, all patients were established on selexipag at 200 mcg twice per day, with dose increments every two–four days by 200 mcg/dose until the maximum achievable dose. We found dosing to be in line with the adult literature at 20–30 mcg/kg every twelve hours. At twelve months, the median maximum dose was 30.0 (*Q*_1_ = 26.0, *Q*_3_ = 36.5) mcg/kg/dose. The maximal therapy was based on previous adult literature (16), the GRIPHON study (9), and early pediatric data (Supplementary Table S1). If a patient developed side effects prior to achieving the target maximum dose, no further dose increases were implemented.

Additional therapy in the form of bilevel positive pressure ventilation (BiPAP) was required by four (16.7%) patients at twelve months. Twelve (50.0%) patients required oxygen therapy at twelve months. By the end of the study period, thirteen (54.2%) patients were on diuretic therapy and four (16.7%) required anticoagulant therapy. Over the study period, there was a reduction in the number of patients on prostacyclin therapy from eleven (45.8%) at baseline to three (12.5%) at twelve months as a third agent. Intravenous/subcutaneous remodulin was the preferred agent. Failing patients were placed back on intravenous/subcutaneous remodulin. Oral and inhaled prostacyclin was not used in the population. Gastrointestinal disturbances were reported in ten (41.7%) patients, cardiovascular adverse effects in three (12.5%), and dry lips or rashing in two (8.3%). Two (8.3%) patients experienced neurological symptoms, namely, headache and mood alteration. See Supplementary Table S5 for a detailed breakdown of adverse effects. Over the course of the study, one patient was delisted for a lung transplant due to disease progression and was kept on triple therapy. This patient survived.

**Figure 1:**
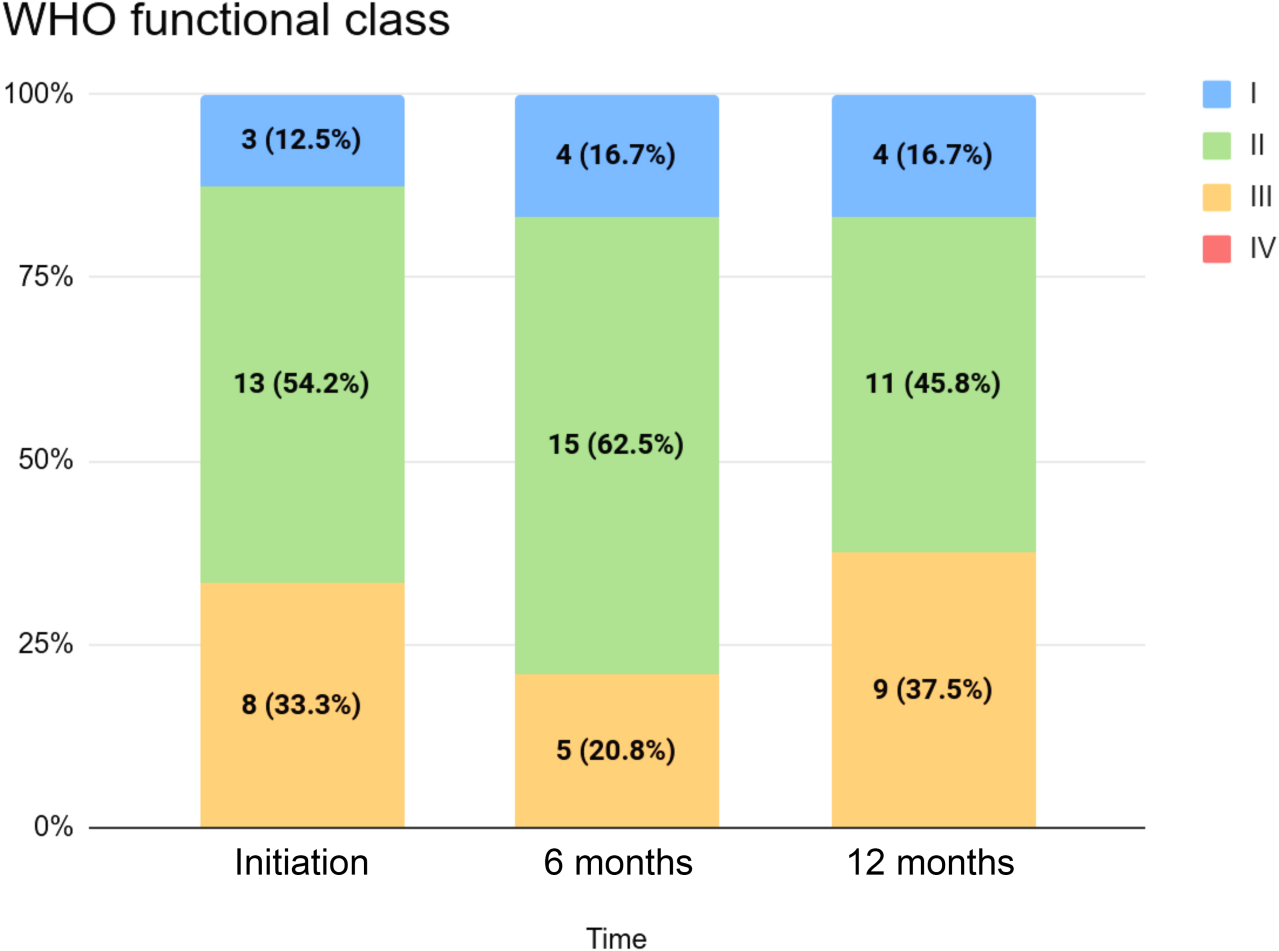
WHO functional class for the cohort at initiation, six months, and twelve months.

Table 2 summarizes clinical and echocardiographic assessments as well as WHO functional classifications (WHO FCs) for the cohort at initiation, six months, and twelve months. The distribution of WHO FC at these time points is visualized in Figure 1. We did not detect statistically significant changes in echocardiographic measures between selexipag initiation and twelve months, although median decreases in tricuspid annular plane systolic excursion (TAPSE), right-ventricular systolic pressure (RVSP), and mean pulmonary arterial pressure (mPAP) were observed. A statistically significant change in six-minute walk test distance (6MWD) was not detected despite an observed increase. Statistically significant changes in WHO FC between initiation and twelve months were not detected: in our cohort, four (16.7%) patients improved with respect to WHO FC, while fifteen (62.5%) had no change and five (20.8%) saw an increase in WHO FC (i.e., a progression of symptoms) over this period.

**Table 2:** Clinical and echocardiographic imaging measures and wHO FC, presented as median (*Q*_1_, *Q*_3_) and count (%), respectively.

Measure	Initiation	6 months	12 months	Comparison^1^
**Echocardiographic measures**				
Eccentricity index	1.5 (1.2, 2.0)	1.4 (1.1, 1.8)	1.3 (1.1, 1.6)	0.0 (-0.3, 0.1) [0.48]
mPAP (mmHg)	40.0 (27.4, 46.6)	20.5 (8.0, 44.5)	24.0 (15.5, 38.0)	-6.1 (-8.7, -3.0) [0.09]
RV/LV	1.0 (0.7, 1.6)	1.1 (0.9, 1.4)	1.1 (1.0, 1.6)	0.0 (-0.1, 0.3) [0.63]
RVFAC (%)	34.0 (28.0, 38.0)	34.5 (31.0, 40.0)	36.0 (29.6, 40.5)	0.0 (-2.0, 10.0) [0.67]
RVSP (mmHg)	73.0 (56.5, 92.5)	58.0 (51.0, 85.0)	64.0 (50.5, 70.0)	-3.5 (-12.5, 16.4) [0.95]
TAPSE (cm)	1.9 (1.6, 2.3)	2.1 (1.5, 2.5)	1.5 (1.2, 1.8)	-0.2 (-0.4, 0.2) [0.17]
TR velocity (cm/s)	387.0 (312.0, 488.6)	280.0 (5.0, 423.0)	302.4 (32.2, 369.1)	-9.6 (-45.7, 0.0) [0.07]
**Six-minute walk test**				
Total distance walked (m)	483.5 (401.8, 571.2)	528.0 (499.0, 619.5)	605.0 (476.2, 623.2)	71.5 (-38.0, 250.0) [0.44]
Pre SaO_2_ (%)	97.0 (91.0, 98.0)	97.0 (95.0, 97.5)	96.0 (94.0, 97.0)	0.0 (-2.5, 1.5) [0.83]
Post SaO_2_ (%)	90.0 (79.0, 93.0)	90.0 (89.0, 90.5)	95.0 (85.0, 97.0)	4.0 (-3.0, 6.0) [0.81]
**WHO FC**				
Class I	3 (12.5%)	4 (16.7%)	4 (16.7%)	0.0 (0.0, 0.0) [1.00]
Class II	13 (54.2%)	15 (62.5%)	11 (45.8%)	
Class III	8 (33.3%)	5 (20.8%)	9 (37.5%)	

Note. In all summaries, *n* ≥ 13 (but *n* = 9 for mPAP and *n* = 24 for WHO functional classification).

Note. RV/LV, ratio of right to left ventricle diameter; RVFAC, right-valve fractional area change; TR, tricuspid regurgitation.

^1^ The Comparison column presents paired differences between initiation and twelve months as median (*Q*_1_, *Q*_3_) [*p*-value]. *P*-values are computed using paired Wilcoxon signed rank tests and are unadjusted. In all tests, *n* ≥ 12 (but *n* = 6 for total distance walked and mPAP, *n* = 7 for pre SaO_2_, and *n* = 5 for post SaO_2_).

Supplementary Table S6 summarizes hemodynamic cardiac catheterization data collected at all four time points. This data is sparse, with measures from at most four patients available at any point, so no formal comparisons were conducted and we do not discuss these results in detail here. In short, we observed median increases in all measures under three different conditions (room conditions with 21% FiO_2_, 100% O_2_, and 100% O_2_ with 20 ppm inhaled NO).

## 4. Discussion

### 4.1. Role of selexipag in treating pediatric PH

While PH is currently a progressive and fatal disease, the treatment landscape is changing rapidly. The delivery of multimodal pharmacotherapy that interacts with different pathways has changed treatment strategies and the clinical course of the disease for many children. Selexipag offers physicians the opportunity to escalate treatment for severe pediatric PAH patients without the challenges associated with central access, compliance to inhalation therapy, or the pain of subcutaneous therapy (site-related or dermatitis). The problem of patient selection aside, it is difficult to ensure that pediatric patients are on equivalent or optimal doses of therapy to prevent disease progression or clinical worsening while minimizing adverse effects.

We recognize that identifying pediatric PH patients who would be most responsive to selexipag therapy is difficult with either noninvasive (e.g., echocardiography) or invasive (e.g., cardiac catheterization) tools. Responses from individual patients are difficult to establish without a trial period. It would be ideal to add selexipag therapy to treatment regimens both prior to disease progression and when patients are most clinically responsive to selexipag: this can only be achieved through a better understanding of the relationship between selexipag's pharmacokinetic and pharmacodynamic properties in the pediatric population. In our current work it is evident that there are specific patients for whom the addition of selexipag therapy to dual combination therapy was beneficial. On a larger scale, timing selexipag therapy for pediatric PH patients requires multicenter collaborations to identify patients likely to respond to selexipag therapy prior to disease progression or deterioration.

### 4.2. Clinical response to selexipag

Overall, we observed improvements in WHO FC, 6MWD, right–left ventricle (RV/LV)diameter ratio, mPAP, and TAPSE, although none of these changes were statistically significant. A prospective study by Hansmann et al. (17) with fifteen patients (age 7 months–17 years) on selexipag observed improvements in mPAP, right-ventricular systolic function, and functional classifications as well as pediatric PH prognostic risk scores that trended toward lower serum NT-proBNP concentrations. In their cohort, three patients showed disease progression and two ultimately received a lung transplant. The authors reported that the efficacy of selexipag was variable but often saw better responses in “less-sick patients”.

Parameters such as WHO FC, RV/LV diameter ratio, and TAPSE have been used as surrogates for transplant-free survival. In the pediatric population, these clinical and imaging parameters assess response to treatment and guide treatment modifications (18). We observed statistically nonsignificant improvements in cardiac catheterization measures, echocardiographic parameters, and clinical outcomes (including WHO FC and 6MWD). In nineteen (79.2%) patients we observed a constant or improved WHO FC over the twelve-month study period. While attempts were made in our cohort to measure serial laboratory parameters, not all patients had their serial NT-proBNP levels drawn. We postulate that clinical improvements in WHO FC and 6MWD are correlated with improvements in NT-proBNP levels and that the former can be used as a treatment response indicator. Research by Hansmann et al. (17) noted that those most likely to respond to selexipag might not all have the same disease severity despite worsening clinical symptoms. Hence, there is a poor correlation between individual disease severity and response to selexipag therapy. Change in individual patients needs to be further delineated to determine criteria for ideal candidates and the optimal timing for initiating therapy.

While an adverse effect profile can be rate-limiting for many clinicians when considering dose or treatment escalation, we chose to gradually optimize doses based on individual patient tolerance and the adverse effects experienced by our cohort (16). Despite slow dose increases, a total of ten (41.7%) patients reported gastrointestinal adverse effects (namely, abdominal pain, decreased appetite, diarrhea, and nausea), three (12.5%) reported cardiac adverse effects (namely, flushing), two (8.3%) reported neurological adverse effects, and two (8.3%) reported dermatological adverse effects. With the exception of one patient who required admission to a pediatric intensive care unit, the patients were managed conservatively and without the need to cease therapy. The tolerability of adverse effects is an imperative consideration when treating pediatric PAH in order to reduce noncompliance and disease progression. Our work suggests that the adverse effects of selexipag are tolerable for patients within the range of the clinical, echocardiographic, and catheterization parameters and at the dose described.

### 4.3. Selexipag for PAH-associated CHD

The early experience of Koestenberger and Hansmann (19) regarding patients with idiopathic PAH and PAH-CHD highlighted the threefold benefit of adding selexipag to standard dual (PDE5 inhibitor and ERA) therapy: it avoids CVL insertion for children and teenagers, helps stabilize the disease, and acts as a bridge to lung transplant. Even when underlying CHD is managed with shunt closure, aggressive therapy for underlying PAH is required to negate the impact of PAH on the pulmonary vascular bed. This is particularly relevant in our study, where seventeen (63.0%) patients had underlying CHD, of whom ten had PAH-CHD. Further research is needed to delineate the role of selexipag in the treatment and management of PAH-CHD and pediatric PH.

The proportion of patients who later develop PAH differs between those with simple and complex CHD (17, 19, 20). Approximately 3% of patients with simple lesions with a small defect (e.g., ventricular septal defects) will develop irreversible damage (i.e., Eisenmenger syndrome) if left untreated. It appears that patients with either simple or complex lesions (the latter nearly always associated with irreversible damage) stand to benefit when selexipag is added to their treatment regimen. Future work can explore the benefits of oral prostanoid therapy for simple or complex CHD and potential reductions in the impact of late-development PAH. Long-term, multicenter pediatric studies are required to better understand the impact of selexipag on PAH-CHD and the utility of this therapy in managing PAH related to simple and complex cardiac lesions.

### 4.4. Dosing, transition strategy, and adverse effects of selexipag

Dosing in PH therapy in general, let al.one with selexipag, has always presented a challenge: the difficulty lies in determining optimal dose targets and in timing therapies as current pediatric dosing is based on the adult literature. For selexipag, this is a maximum of 1,600 mcg twice per day or until prostacyclin-managed side effects cannot be managed (16). Even in the adult population, dosing is not clear: the median dose in the FREEDOM-M trial following twelve weeks of gradual increases was 3,400 mcg twice daily with a comment that the “maintenance dose is determined by tolerability” (17, 20). As previously mentioned, other works (16) have proposed a maximum dose of 1,600 mcg twice daily with the caveat that the maximum dose should be tailored to the tolerability of adverse effects. In our multicenter study, all participants were titrated to twice-daily doses of 20–30 mcg/kg/dose as per the adult literature. Most patients who weighed more than 45 kg maxed out at twice-daily doses of 1,400 mcg due to side effects, similar to what is described in the adult experience. Our study was not designed to determine the pharmacokinetics of selexipag in a heterogeneous group of PAH patients. As such, more collaborative work is needed to determine optimal dosing for patients with pediatric PAH.

The dosing regimen employed in this work adhered to the following points.

• Children weighing under 20 kg were started on a twice-daily, 100 mcg regimen, with increases of 100 mcg/dose every week up to a target dose of 30 mcg/kg twice daily.
• Children weighing more than 20 kg were started at 200 mcg twice daily with increases of 200 mcg/dose each week to a target dose of 30 mcg/kg twice daily with a maximum dose of 1,600 mcg twice daily.

Kanaan et al. (21) implemented a different dosing schedule with two-thirds of their cohort on a median dose of 2,000 mcg/dose three times daily. This regimen may have been implemented to facilitate the transition from intravenous or subcutaneous treprostinil therapy to enteral selexipag and may account for the adverse effects noted in their cohort. Hansmann et al. (17) implemented dosing similar to that in our study that aimed for a final dose of 30 mcg/kg/dose twice daily. The jaw and neck pain reported in the GRIPHON study (by 17%–26% of patients) was not found in our cohort over the study period: this could be related to the medical interpretation of reports from children. The most frequently reported adverse effects in our cohort were gastrointestinal: three patients had abdominal pain, three had decreased appetite, three had diarrhea, and one had nausea. Side effects were managed conservatively and no patients ceased therapy secondary to these effects.

#### 4.4.1. Transition strategy

Transitions from intravenous or subcutaneous treprostinil were performed over the course of four–seven days in hospital by titrating down treprostinil with each dose of selexipag and generally escalating each dose by 100–200 mcg depending on patient weight (100 mcg increases for patients under 20 kg and 200 mcg increases for patients over 20 kg) to a goal of 30 mcg/kg/dose twice per day or a maximum of 1,600 mcg twice daily (although as above, most patients were stopped at a twice-daily, 1,400 mcg dose due to side effects). If patients experienced adverse effects from excessive vasodilator side effects (e.g., headache, flushing, dizziness, low blood pressure), the selexipag dose was held constant for one or two titrations down on remodulin. Most titrations of remodulin were in 2.5–5.0 ng/kg/min increments. The site was generally kept running low-dose normal saline for 24 h after achieving the target selexipag dose to ensure that a re-initiation of remodulin was not necessary. All but one transition was performed in hospital. One home transition was performed with weekly titrations following a similar pattern.

The difference in adverse effects reported by adults and children can likely be explained at the cellular level. In addition to metabolic differences, there are characteristic differences in receptor numbers and density between adults and children (22). Through the action of prostanoid receptors on the endogenous prostacyclin pathway, variability in the number of receptors and their responsiveness may account for differences in the frequency and number of adverse effects reported by adults and children as well as differences in treatment efficacy between these populations. However, there is currently no way to use prostacyclin receptor numbers to characterize patients with a robust or minimal response to selexipag.

Additionally, predisposing genetic syndromes in the pediatric population need to be considered: up to 34% of individuals with Down syndrome and PH are known to have gastroesophageal reflux disease and thus a possible predisposition to gastrointestinal upsets and aspiration (23). Further exploration of optimal dosing, pharmacokinetics, and pharmacodynamics in pediatric PAH is necessary to minimize adverse effects and maximize drug efficacy (19, 23).

### 4.5. Patient selection

One common theme in our experience is the lack of a clear selection procedure to identify subgroups of pediatric PH patients that would benefit most from the addition of selexipag and the disease stage at which an oral agent acting on the prostacyclin pathway would be most effective. We utilized selexipag therapy in three broad groups. The first included patients who were slowly improving (with respect to WHO FC, hemodynamics, and 6MWD) and prostacyclin naïve (*n* = 5). The second contained patients further along in the course of the disease who improved on intravenous or subcutaneous prostacyclin therapy but wanted to try an oral agent for quality-of-life reasons (*n* = 11). The third group contained a small subset of patients for which selexipag was introduced on compassionate grounds where intravenous or subcutaneous prostacyclin was not an option: this was most commonly for behavioral reasons and a family's or caregiver's strong belief, after weighing respective risks and benefits, that an oral medication was favorable (*n* = 8). In these cases, doses were occasionally pushed higher than the standard 20–30 mcg/kg/dose in the twice-daily regime.

The lack of knowledge on whether selexipag has the same benefits as traditional prostacyclin in terms of remodeling with antiproliferation and antiplatelet action presents another significant challenge. This therapy is still in its infancy for the pediatric population, so this question will remain unanswered until more long-term data is collected.

### 4.6. Limitations

We recognize that our multicenter data is retrospective and dependent on chart reviews of a small (but, relative to previous works, notable) number of pediatric PAH patients. We are not able to comment on long-term follow-up for these patients given the cohort's heterogeneity, the death of three patients, and the need for one patient to be listed for lung transplant. While we observed patients who benefited from selexipag therapy, we also observed patients with no change despite treatment with maximally tolerable therapy. Further collaborative work to determine criteria for identifying ideal candidates (i.e., those who respond to early selexipag therapy) is required. Additionally, our study cohort did not have biochemical data (e.g., serial measurements of NT-proBNP) available for all patients as surveillance protocols differed between the three centers.

## 5. Conclusion

Selexipag, an oral prostanoid with an active metabolite, offers clinicians a promising therapeutic option for escalating treatment in pediatric patients with severe PH without the challenges associated with other current therapies. In this work, we presented a multicenter experience with this therapy in a heterogeneous group of pediatric PAH patients. A number of patients benefited from the addition of selexipag to standard dual (PDE5 inhibitor and ERA) therapy. Statistically significant changes in echocardiographic parameters, hemodynamic measures, and WHO FC were not detected over the twelve-month study period, although we observed improved or constant WHO FCs for nineteen (79.2%) patients. Nonetheless, we argue that this therapy is an invaluable addition to current treatment regimens, especially for patients who are not suitable candidates for intravenous or subcutaneous therapy and who are unlikely to comply with multiday inhalation therapy or for whom disease stabilization is required as a bridge to transplant. The most common adverse effects of this therapy are gastrointestinal in nature. Dosing should reach the current literature's recommendation of 30 mcg/kg/dose twice per day with adjustments according to adverse effects. Future work should focus on the relationship between selexipag's pharmacokinetics and pharmacodynamics in pediatric PH patients to determine optimal initiation and dosing regimens, maximize adherence, and minimize intolerance.

## Data Availability

The original contributions presented in the study are included in the article/**Supplementary Materials**. Further inquiries can be directed to the corresponding author.
