# Supplementary material for: A Canadian, retrospective, multicenter experience with selexipag for a heterogeneous group of pediatric pulmonary hypertension patients
Source: Front Pediatr. 2023 Feb 28;11:1055158. doi: 10.3389/fped.2023.1055158 (PMC10011093; doi:10.3389/fped.2023.1055158)
Supplement: Supplementary file 1 [file Datasheet1.pdf]

Supplementary Material

1 Supplementary Tables

Supplementary Table 1. Summary of Current Literature on the Use of Selexipag to Treat Pediatric PH

Reference (design)	Number of patients	Treatment duration	Endpoints/outcome	Additional pulmonary vasodilator therapy
GRIPHON (2015)<br>(randomized, double-blind, placebo-controlled study)<br>(1)	*n* = 1156 adults (574 on selexipag)	70.7 weeks	- 40% decrease in morbidity and mortality (death from any cause or complication related to PAH)<br>- 0.60 hazard ratio for a primary endpoint event in the selexipag group<br>- Addition of selexipag to dual (ERA+PDE5i) therapy reduced rate of death or secondary complications related to PAH	PDE5i ± ERA
Gallotti et al. (2017)<br>(case series)<br>(2)	*n* = 10 (age range 7–20 years, 4 transitioned from intravenous treprostinil)	Unclear	- All remained clinically stable according to echo and 6MWT<br>- 1 patient weaned off daytime oxygen<br>- 1 patient reported increased exercise capacity	- PDE5i + ERA<br>- 4 patients transitioned from intravenous treprostinil to oral selexipag
Geerdink et al. (2017)<br>(case report)<br>(3)	*n* = 1 (12-year-old female with heritable PAH)	6 months	- Hemodynamic and clinical improvements<br>- 5 deaths unrelated to selexipag	PDE5i + ERA
Davis and Yung (2018)<br>(case series)<br>(4)	*n* = 3 (7 years old, trisomy 21, PAH-CHD, bosentan and tadalafil; 2 years old, single ventricle physiology, sildenafil; 6 years old, Eisenmenger syndrome, single-agent tadalafil)	5 months	- At 1 month, 2 patients had improved O_2 saturations and 1 patient had improved endurance<br>- At 1 month, 1 patient was unchanged<br>- Patient 1 had diarrhea, emesis, body aches; patient 2 had no side effects; patient 3 had diarrhea	PDE5i ± ERA
Kanaan et al. (2019)<br>(multicenter, retrospective observational study)<br>(5)	*n* = 28 across 4 pediatric PH programs<br>(median age 6.5 years)	2 years	- 6 prostanoid naïve and 22 transitioned from another prostanoid to oral treprostinil<br>- 1 patient treated with selexipag died: investigators do not feel this was secondary to selexipag failure<br>- Median total daily dose for the 14 patients still taking oral treprostinil was 9 (Q_1 = 5, Q_3 = 12) mg	- PDE5i ± ERA<br>- 14 patients discontinued medication due to gastrointestinal side effects after 2 years<br>- Gastrointestinal symptoms (nausea, vomiting, diarrhea) accounted for >50% of complaints, followed by headaches in 15%
Koo et al. (2019)<br>(case report)<br>(6)	*n* = 1 (11.5 months old, 8.6 kg, converting from subcutaneous treprostinil	12 months	- Echo at 12-month follow-up showed mild pulmonary hypertension	Transitioned from intravenous treprostinil to selexipag

	40 ng/kg/min to selexipag)		- Repeat NT-proBNP remained low	
Hansmann et al. (2020) (prospective observational study) (7)	- *n* = 15 across 3 centers (age 7 months–17 years, PAH and precapillary PH, oral add-on selexipag, 2 lung transplants)<br>- Excluded patients with WHO FC class IV, intra- or extra-cardiac shunts	Median 8 months	- 1 patient died while on treprostinil and not actively on selexipag<br>- Improvement in mean RAP, PAP/SAP, TPG, TAPSE, WHO FC<br>- Outcomes improved in 50% and disease progression was prevented in 27%	- PDE5i ± ERA<br>- Main side effects were nausea, headache, vomiting, jaw pain (17%–26%)
Rothman et al. (2020) (case series) (8)	*n* = 4 (age 5 months–11 years)	12–18 months	- Selexipag used as a substitute for subcutaneous treprostinil for 1 patient<br>- Selexipag added as a third agent for 3 patients due to haemodynamic deterioration<br>- Cardiac catheterisation showed no change in PVRI: small decrease in 3 patients, small increase in 1 patient	- PDE5i ± ERA<br>-Gastrointestinal symptoms including diarrhea, headaches, jaw pain.
Lafuente-Romero and Rodriguez Ogando (2021) (case series) (9)	*n* = 4 (ages 21 months and 6, 12, 21 years; 2 pulmonary atresia +VSD+MAPCA; 1 trisomy 21+AVSD)	12–39 months	- Clinical benefit of selexipag allowed weaning nighttime oxygen for 1 patient and discontinuation of epoprostenol for 1 patient<br>- 2 patients remained stable	PDE5i + ERA

Note. 6MWT, six-minute walk test; AVSD, atrioventricular septal defect; CHD, congenital heart disease; ERA, endothelin receptor antagonist; MAPCA, major aortopulmonary collateral artery; NT-proBNP, N-terminal pro B-natriuretic peptide; PA, pulmonary artery; PAH, pulmonary arterial hypertension; PAP/SAP, pulmonary arterial pressure/systolic arterial pressure; PDE5i, phosphosphodiesterase type 5 inhibitor; PH, pulmonary hypertension; PVRI, pulmonary vascular resistance index; RAP, right artery pressure; TAPSE, tricuspid annular plane systolic excursion; TPG, transpulmonary gradient; VSD, ventricular septal defect; WHO FC, WHO functional classification

Supplementary Table 2. Comorbidity Classification at Selexipag Initiation, Presented as Count (%)

Comorbidity/subclassification	Count (%)
**Coagulation disorder**	**1 (4.2%)**
Factor V Leiden mutation	1 (4.2%)
**ENT**	**5 (20.8%)**
Cleft lip and palate	1 (4.2%)
Conductive hearing loss	1 (4.2%)
Laryngeal cleft	1 (4.2%)
Recurrent epistaxis	1 (4.2%)
Sleep-disordered breathing	1 (4.2%)
**Gastrointestinal**	**1 (4.2%)**
Situs inversus totalis	1 (4.2%)
**Musculoskeletal**	**2 (8.3%)**
Osteogenesis imperfecta	1 (4.2%)
Scoliosis	1 (4.2%)
**Neurological**	**1 (4.2%)**
Periventricular white matter	1 (4.2%)
**Obesity**	**1 (4.2%)**
**Preterm infant**	**2 (8.3%)**
**Respiratory**	**3 (12.5%)**
Chronic lung disease	1 (4.2%)
Congenital lymphangiectasia	1 (4.2%)
History of aspiration	1 (4.2%)
**None**	**9 (37.5%)**

Supplementary Table 3. Anthropometry at Each Time Point, Presented as Median (Q_1, Q_3)

Variable	Initiation	6 months	12 months
Height (cm)	136.0 (112.9, 149.6)	139.5 (135.0, 149.8)	140.8 (135.5, 153.0)
Weight (kg)	28.4 (24.4, 37.7)	30.4 (26.9, 38.9)	32.0 (27.2, 43.2)
Body surface area (m^2)	1.0 (0.8, 1.3)	1.1 (1.0, 1.3)	1.1 (1.0, 1.4)

Note. At each time point, $n \geq 23$ observations are used.

Supplementary Table 4. Pulmonary Vasodilator Therapy and Additional Therapy at Each Time Point, Presented as Median (Q_1, Q_3) or Count (%)

Variable	Baseline	Initiation	6 months	12 months
Selexipag				
Maximum dosage (mcg/kg/dose)	-	19.0 (7.5, 24.0)	27.5 (21.0, 36.2)	30.0 (26.0, 36.5)
Other medication				
Endothelin receptor antagonist	24 (100.0%)	24 (100.0%)	22 (91.7%)	22 (91.7%)
Phosphodiesterase type-5 inhibitor	24 (100.0%)	23 (95.8%)	22 (91.7%)	22 (91.7%)
Prostacyclin	11 (45.8%)	10 (41.7%)	4 (16.7%)	3 (12.5%)
None	0 (0.0%)	0 (0.0%)	0 (0.0%)	0 (0.0%)
Other therapy				
Aspirin	3 (12.5%)	2 (8.3%)	1 (4.2%)	1 (4.2%)
BiPAP/CPAP	2 (8.3%)	3 (12.5%)	3 (12.5%)	4 (16.7%)
Calcium channel blocker	0 (0.0%)	0 (0.0%)	0 (0.0%)	0 (0.0%)
Clopidogrel	0 (0.0%)	0 (0.0%)	0 (0.0%)	0 (0.0%)
Coumadin	2 (8.3%)	2 (8.3%)	3 (12.5%)	3 (12.5%)
Diuretics	14 (58.3%)	12 (50.0%)	10 (41.7%)	13 (54.2%)
Oxygen	13 (54.2%)	13 (54.2%)	10 (41.7%)	12 (50.0%)
Thyroid replacement	3 (12.5%)	2 (8.3%)	0 (0.0%)	1 (4.2%)
Other	5 (20.8%)	5 (20.8%)	3 (12.5%)	2 (8.3%)

Note. BiPAP, bilevel positive airway pressure; CPAP, continuous positive airway pressure

Supplementary Table 5. Number of Patients (%) with Adverse Effects at any Time After Initiation, Stratified by System

System (adverse effect)	Count (%)
**Cardiovascular**	**3 (12.5%)**
Dizziness	0 (0.0%)
Flushing	3 (12.5%)
Hypotension	0 (0.0%)
Presyncope	0 (0.0%)
**Dermatological**	**2 (8.3%)**
Dry lips	1 (4.2%)
Rash	1 (4.2%)
**Gastrointestinal**	**10 (41.7%)**
Abdominal pain	3 (12.5%)
Decreased appetite	3 (12.5%)
Diarrhea	3 (12.5%)
Nausea	1 (4.2%)
Reflux	0 (0.0%)
Vomiting	0 (0.0%)
**Musculoskeletal**	**0 (0.0%)**
Back pain	0 (0.0%)
Jaw pain	0 (0.0%)
Leg pain	0 (0.0%)
**Neurological**	**2 (8.3%)**
Headache	1 (4.2%)
Mood alteration	1 (4.2%)
**Respiratory**	**1 (4.2%)**

Decreased exercise tolerance	0 (0.0%)
Decreased oxygen saturation	1 (4.2%)
Shortness of breath	0 (0.0%)

Supplementary Table 6. Cardiac Catheterization and Hemodynamic Measures at Each Time Point, Stratified by Environment and Presented as Median (Min–Max)

Property	Baseline	Initiation	6 months	12 months
Condition: room conditions, 21% FiO_2				
CI ($L/min/m^2$)	3.7 (2.8–5.4)	4.3 (3.1–5.5)	3.8 (3.8–3.8)	4.1 (2.8–5.1)
mPAP (mmHg)	47.0 (24.0–71.0)	53.0 (42.0–63.0)	49.0 (49.0–49.0)	43.0 (33.0–49.0)
PVR/SVR	0.7 (0.3–1.6)	2.0 (0.5–3.5)	0.7 (0.7–0.7)	0.7 (0.3–0.8)
PVRI (WU)	8.4 (4.0–20.9)	12.0 (7.4–14.0)	-	8.0 (5.0–15.1)
RAP (mmHg)	6.0 (4.0–11.0)	7.0 (6.0–18.0)	-	8.0 (5.0–10.0)
RVSP (mmHg)	64.0 (33.0–90.0)	77.5 (71.0–84.0)	-	59.0 (48.0–69.0)
Condition: 100% O_2				
CI ($L/min/m^2$)	3.6 (2.4–4.6)	3.0 (2.9–3.1)	-	4.3 (2.8–5.3)
mPAP (mmHg)	60.0 (48.0–78.0)	45.0 (37.0–47.0)	-	33.0 (32.0–45.0)
PVR/SVR	0.7 (0.5–1.0)	0.7 (0.5–0.9)	-	0.4 (0.3–0.7)
PVRI (WU)	11.4 (7.6–20.4)	10.0 (7.1–12.9)	-	6.1 (5.0–13.9)
RAP (mmHg)	7.0 (4.0–9.0)	7.0 (7.0–7.0)	-	6.0 (5.0–9.0)
RVSP (mmHg)	90.0 (90.0–90.0)	87.5 (70.0–105.0)	-	51.0 (51.0–51.0)
Condition: 100% O_2 and 20 ppm inhaled NO				
CI ($L/min/m^2$)	3.5	2.9	-	4.1

	(2.5–4.8)	(2.8–5.5)		(2.6–5.3)
mPAP (mmHg)	44.0 (19.0–62.0)	43.0 (37.0–53.0)	50.0 (50.0–50.0)	36.0 (30.0–44.0)
PVR/SVR	0.5 (0.3–1.0)	0.6 (0.3–3.5)	-	0.4 (0.3–0.8)
PVRI (WU)	8.8 (2.3–20.0)	10.9 (58.8–14.0)	-	5.5 (5.0–12.7)
RAP (mmHg)	7.5 (2.0–9.0)	7.0 (5.0–18.0)	-	6.5 (4.0–10.0)
RVSP (mmHg)	60.0 (28.0–81.0)	61.5 (61.0–62.0)	-	46.0 (45.0–61.0)

Note. "-" denotes the absence of observations. In other cells, $n \leq 4$ observations are used in the summary at each time point.

Note. CI, cardiac index; mPAP, mean pulmonary arterial pressure; PVR/SVR, ratio of pulmonary vascular resistance to systemic vascular resistance; PVRI, pulmonary vascular resistance index; RAP, right artery pressure; RVSP, right-ventricular systolic pressure; WU, Wood units

References

1. Sitbon O, Channick R, Chin KM, Frey A, Gaine S, Galiè N et al. Selexipag for the treatment of pulmonary arterial hypertension. New England Journal of Medicine (2015) 373(26):2522–33.

2. Gallotti R, Drogalis-Kim DE, Satou G, Alejos J. Single-center experience using selexipag in a pediatric population. Pediatric Cardiology (2017) 38(7):1405–9.

3. Geerdink LM, Bertram H, Hansmann G. First-in-child use of the oral selective prostacyclin IP receptor agonist selexipag in pulmonary arterial hypertension. Pulmonary Circulation (2017) 7(2):551–4.

4. Davis A, Yung A. Selexipag in three pediatric patients. In: Abstracts from the 11th International Conference on Neonatal and Childhood Pulmonary Vascular Disease 2018 Apr 19–21; San Francisco, CA, USA. Pulmonary Circulation (2018) 8(3):2045894018782405.

5. Kanaan U, Varghese NP, Coleman RD, Huckaby J, Lawrence P, Jorgensen LO et al. Oral treprostinil use in children: A multicenter, observational experience. Pulmonary Circulation (2019) 9(3):2045894019862138.

6. Koo R, Lo J, Bock MJ. Transition from intravenous treprostinil to enteral selexipag in an infant with pulmonary arterial hypertension. Cardiology in the Young (2019) 29(6):849–51.

7. Hansmann G, Meinel K, Bukova M, Chouvarine P, Wåhlander H, Koestenberger M et al. Selexipag for the treatment of children with pulmonary arterial hypertension: First multicenter experience in drug safety and efficacy. The Journal of Heart and Lung Transplantation (2020) 39(7):695–6.

8. Rothman A, Cruz G, Evans WN, Restrepo H. Hemodynamic and clinical effects of selexipag in children with pulmonary hypertension. Pulmonary Circulation (2020) 10(1):2045894019876545.

9. Lafuente-Romero A, Rodriguez Ogando A. Selexipag use for pediatric pulmonary hypertension: A single center report focussed on congenital heart disease patients. Cardiology in the Young (2021) 31(9):1513–5.
